# Nutrient-Derived Dietary Patterns and Their Association With Metabolic Syndrome in a Japanese Population

**DOI:** 10.2188/jea.JE20170010

**Published:** 2018-04-05

**Authors:** Tirani Bahari, Hirokazu Uemura, Sakurako Katsuura-Kamano, Miwa Yamaguchi, Mariko Nakamoto, Keisuke Miki, Masashi Ishizu, Kokichi Arisawa

**Affiliations:** 1 Department of Preventive Medicine, Institute of Biomedical Sciences, Tokushima University Graduate School, Tokushima, Japan; 2 Department of Public Health and Applied Nutrition, Institute of Biomedical Sciences, Tokushima University Graduate School, Tokushima, Japan

**Keywords:** dietary pattern, reduced rank regression, nutrients, metabolic syndrome

## Abstract

**Background:**

Nutrients have been proposed to be related to metabolic syndrome (MetS). The aims of this study were to identify dietary patterns that correlated with several nutrients using reduced rank regression (RRR) and to examine the association between extracted dietary patterns and prevalence of MetS in a Japanese population.

**Methods:**

The study population comprised 1,092 Japanese men and women (35–69 years old) who had participated in the baseline survey of the Japan Multi-Institutional Collaborative Cohort Study in Tokushima Prefecture. Dietary patterns were derived with RRR using 46 food items as predictors and six established nutrients (potassium, calcium, vitamin D, vitamin C, insoluble dietary fiber, and carotene) as response variables. Associations between extracted dietary patterns and MetS were then examined with logistic regression models.

**Results:**

Among the six dietary patterns, dietary pattern 1 (DP1) explained the largest proportion (60.1%) of variance in the six nutrients. Therefore, only DP1 was selected for further analysis. DP1 was characterized by high intake frequency of vegetables, fruits, fish and small fish, natto (fermented soybeans), and deep-fried tofu. After adjustment for potential confounders, significant inverse associations were found between DP1 score and MetS (odds ratio [OR] for each quartile: 1.00, 0.58, 0.60, 0.52; *P*_trend_ = 0.02); DP1 and high blood pressure (*P*_trend_ = 0.0002); and DP1 and high blood glucose (*P*_trend_ = 0.02).

**Conclusion:**

A dietary pattern characterized by high intake of vegetables, fruits, fish and small fish, natto, and deep-fried tofu was associated with reduced prevalence of MetS in a Japanese population.

## INTRODUCTION

Metabolic syndrome (MetS) is characterized by abdominal obesity, insulin resistance, dyslipidemia, hypertension, and glucose intolerance.^1^ People with MetS are at increased risk of type 2 diabetes mellitus and cardiovascular diseases.^2^^–^^4^ The International Diabetes Federation estimates that one-quarter of the adult population in the world have MetS,^5^ while in Japan the prevalence of MetS was estimated at 23.6% for men and 9.1% for women in 2013.^6^ Therefore, a primary prevention of MetS is necessary.

In addition to body mass index (BMI) and physical activity levels,^7^ dietary habits may be closely related to the development of MetS.^8^ Several previous studies have examined the relation between intake of macronutrients and MetS.^9^^,^^10^ Low intake of several other nutrients has also been shown to be related with MetS.^11^ Nutrients such as potassium (K),^12^^,^^13^ calcium (Ca),^14^^,^^15^ vitamin D,^14^ vitamin C,^16^ insoluble dietary fiber (IDF),^17^ and carotene^18^ are reported to be inversely associated with MetS and its components.

The use of dietary patterns has become a tool to examine overall diet consumption because it reflects a more extensive view of foods and nutrients,^19^ and it is generally difficult to observe the effect of a single dietary component due to the high inter-correlation among foods and nutrients.^20^ Currently, there are two methods available to assess the dietary pattern in the population—the hypothesis-oriented method and the exploratory method. The hypothesis-oriented method is based on prior information, such as diet scores, based on current scientific evidence or dietary guidelines. Meanwhile, in the exploratory methods, like Principal Component Analysis (PCA) and factor analysis, the dietary patterns are obtained from the data in the specific population, which aim to explain the proportion of variance in food intake as much as possible.^21^^,^^22^ However, because PCA and factor analysis use no prior information, they may not always extract dietary patterns that are predictive of diseases.^23^

Reduced rank regression (RRR) is a method that generates a linear combination of food groups that explains as much variation as possible in response variables (intermediate variables, such as nutrients and biomarkers), rather than variation in food intake.^23^^,^^24^ It is used to investigate the diet’s variation associated with the variables that are hypothesized to be on the pathway to disease development.^21^^,^^24^ Compared to PCA that only uses data from the specific population, RRR combines the hypothesis-oriented method and exploratory method, so it incorporates the data from prior knowledge to select the response variables and data from the specific population.^22^ Previous studies have shown the advantage of using RRR to derive the dietary pattern and analyze its association with chronic diseases and conditions, such as type 2 diabetes mellitus, stroke, coronary artery disease, and obesity.^21^^,^^25^^–^^27^ However, to the best of our knowledge, there has been no study that applied RRR to examine the association of nutrient-derived dietary patterns with MetS in an Asian population.

The purpose of this study was to identify any dietary patterns that correlated with several different nutrients using RRR and, subsequently, to examine the association between the dietary pattern and the prevalence of MetS in a Japanese population.

## MATERIAL AND METHODS

### Study population

The population for the present study consisted of 1,269 participants, aged 35 to 69 years, who were enrolled in the baseline survey of the Japan Multi-Institutional Collaborative Cohort (J-MICC) Study in Tokushima Prefecture, Japan. Details about the J-MICC Study have been described in a previous report.^28^ In short, the aim of the J-MICC Study was to examine prospectively the associations of lifestyle and genetic factors and their interactions with the risk of chronic diseases. The participants for this study consisted of two recruitment groups. The participants for the first group were people who had attended the Tokushima Prefectural General Health Check-up Center from January 23, 2008 to November 24, 2011. From 3,911 subjects who had received a medical examination during the study period and were asked to participate in the J-MICC study, 573 people agreed to take part in this study. We also distributed approximately 98,700 leaflets explaining the objective and method of the J-MICC Study all over Tokushima city, which has a total population of 264,500. The second group consisted of 696 subjects who read leaflets about this study and attended the health check-ups performed by our research team from July 25, 2012 to February 27, 2013. The participation rate of the second group was difficult to calculate because of the recruitment method.

Written informed consent was obtained from participants after we explained the outline and objectives of this study. The study protocol was approved by the institutional review boards of Nagoya University School of Medicine (an affiliate of former principal investigator, Dr. Nobuyuki Hamajima) (IRB No. 939-13), Aichi Cancer Center Research Institute (affiliated with the present principal investigator, Dr. Hideo Tanaka) (IRB No. 2016-2-10), and Tokushima University Hospital (IRB No. 466-1).

We excluded participants who had history of ischemic heart disease (defined as any angina pectoris or myocardial infarction) (*n* = 29), stroke (*n* = 14), diabetes mellitus (*n* = 81), or who received medical treatment with anti-diabetic drugs (*n* = 50). Participants could be excluded for one or more reasons. Subjects with missing values of other variables (*n* = 24) were also excluded from the study. These exclusions gave a final study population of 1,092 participants (537 men and 555 women) for statistical analysis.

### Questionnaire

In this study, subjects were asked to fill out a self-administered questionnaire about dietary habits, current and previous diseases, medication and supplements consumption, physical activity, and smoking and drinking habits. A validated short food frequency questionnaire (FFQ) was used for dietary evaluations.^29^^–^^32^ Dietary intake information was collected by asking the participants about how often they consumed 46 foods over the past year. Consumption of rice, bread, and noodles at breakfast, lunch, and dinner were divided into six categories: rarely, 1–3 times per month, 1–2 times per week, 3–4 times per week, 5–6 times per week, and every day. Other food items, including coffee and green tea, were categorized into eight categories: rarely, 1–3 times per month, 1–2 times per week, 3–4 times per week, 5–6 times per week, once per day, 2 times per day, and ≥3 times per day. Information on the portion size was collected only for staple foods.

Average daily consumption of energy and selected nutrients were computed using a program developed by the Department of Public Health, School of Medicine, Nagoya City University.^29^^,^^30^ A relative validation study was completed by comparing the intake of energy and 26 nutrients evaluated using this FFQ and 3-day weighed diet records (3d-WDRs) as a reference. Deattenuated, log-transformed, and energy-adjusted Pearson’s correlation coefficients for intake of 26 nutrients distributed from 0.10–0.86.^30^ This FFQ had a very high 1-year interval reproducibility for consumption of foods and nutrients assessment.^32^

Smoking status was self-reported and classified into current smoker, past smoker, and never smoker, while drinking habit was categorized into current drinker, past drinker, and never drinker. Physical activity during leisure time was estimated by multiplying the frequency and average duration of light (such as walking and golf at 3.4 metabolic equivalents [METs]), moderate (such as jogging, swimming, and dancing at 7.0 METs), and vigorous-intensity exercises (such as a marathon at 10.0 METs). The three levels of exercises were summed to obtain the MET-hours/week. Height and weight were also measured, and BMI was calculated as weight (kg) divided by the square of height (m^2^).

### Anthropometric and biochemical measurement

For the subjects who attended the Tokushima Prefectural General Health Check-up Center (the first recruitment group), data on anthropometric measurement (height, weight, and waist circumference), blood pressure, serum triglyceride, high-density lipoprotein (HDL) cholesterol, and fasting plasma glucose were obtained at the time of routine health check-up. Participants were requested not to eat breakfast and received medical check-up between 8.00 AM to 11.30 AM. Venous blood samples were collected from all participants and serum was separated within 3 hours. For the second recruitment group, health check-up was performed by our research team. The procedure used for the health check-up was essentially the same as the first recruitment group.

### Definition of MetS

In this study, the definition of MetS proposed by the National Cholesterol Education Program Adult Treatment Panel III (NCEP ATP III) for Asian populations was used.^33^ Participants with three or more of the following five conditions were defined as having MetS: 1) waist circumference ≥90 cm for men and ≥80 cm for women, 2) blood pressure ≥130/85 mm Hg or hypertension treatment, 3) HDL cholesterol level <40 mg/dL for men and <50 mg/dL for women, 4) triglycerides ≥150 mg/dL, and 5) fasting glucose level ≥100 mg/dL.

### Statistical analysis

Dietary patterns were extracted with RRR using PROC PLS procedure of SAS software (SAS Institute Inc., Cary, NC, USA). This method generates the linear function of food groups that maximally describe variation in response variables.^21^ We chose six nutrients as response variables—K, Ca, vitamin D, vitamin C, IDF, and carotene—because these nutrients are known to be inversely associated with the components of MetS.^13^^,^^14^^,^^16^^–^^18^^,^^34^ Dietary patterns associated with the intakes of these six nutrients were attained based on 46 food items. The number of dietary patterns extracted using RRR was determined by the number of response variables. Correlations between extracted dietary pattern score and nutrients that act as response variables were evaluated using Spearman’s rank correlation coefficients.

Continuous variables of background characteristics were shown as median (25^th^ and 75^th^ percentiles), and categorical variables were expressed as the count and proportion. We examined the differences in characteristics of participants across the quartiles of dietary pattern score using the chi-square test, Fisher’s exact test, or Kruskal-Wallis test.

Multivariate logistic regression analysis was performed to evaluate the associations of dietary pattern with MetS and its components, with adjustment for potential confounding variables, such as age and sex for model 1, and variables included in model 1 plus energy intake (quartiles), physical activity (quartiles), drinking habit (three categories), smoking habit (three categories), and recruitment group (two categories) for model 2. Trends across the quartiles were examined using ordinal categorical variables for dietary pattern score and likelihood ratio test. The interaction by sex on the association between dietary pattern and MetS was examined by including a product term of sex and dietary pattern score in models. As supplementary analysis, logistic regression analysis was performed using sex-specific cut-off points for dietary pattern score (quartiles), energy intake (quartiles), and physical activity (quartiles). All data analyses were conducted using SAS version 8.2.

## RESULTS

Table 1 shows the explained variations in nutrients and food intake, and Spearman’s correlation between nutrients (response variables) and RRR derived dietary patterns. The first pattern was positively correlated with the intake of IDF, K, carotene, vitamin C, Ca, and Vitamin D. The second pattern was positively correlated with Ca and K. For the third pattern, only intake of vitamin D was significantly and positively correlated. The fourth pattern was positively correlated with vitamin C. The fifth pattern was weakly and positively correlated with IDF, carotene, and Ca. The last pattern was positively related with carotene, vitamin C, and Ca. Compared with the other dietary patterns, dietary pattern 1 (DP1) explained by far the largest proportion of variance in selected nutrients, at 60.1%, and explained 14.3% of the variation in food intake. Hence, we selected only DP1 for further analysis.

**Table 1.** Explained variations in nutrients and food intake and Spearman rank correlation between nutrients (response variables) and RRR-derived dietary patterns

	Dietary patterns						Total explained variation

	1	2	3	4	5	6	
Explained variation in food intake, %	14.34	2.87	2.88	2.46	2.43	1.89	26.88
Explained variation in nutrients, %	60.08	12.87	12.39	7.62	4.06	2.34	99.35
**Spearman rank correlation coefficient**							
K	0.84^**^	0.18^**^	−0.11^*^	−0.15^**^	−0.45^**^	−0.08^*^	
Ca	0.64^**^	0.70^**^	0.06^*^	0.12^**^	0.11^*^	0.06^*^	
Vitamin D	0.57^**^	−0.20^**^	0.68^**^	0.02	0.02	−0.02	
Vitamin C	0.79^**^	−0.21^**^	−0.11^*^	0.57^**^	−0.10^*^	0.15^**^	
IDF	0.88^**^	−0.15^**^	−0.19^**^	0.13^**^	0.16^**^	−0.29^**^	
Carotene	0.81^**^	−0.18^**^	−0.22^**^	−0.19^**^	0.14^**^	0.14^**^	

Ca, calcium; IDF, insoluble dietary fiber; K, potassium; RRR, reduced rank regression.

^*^P < 0.05.

^**^P < 0.0001.

Factor loadings of the food intake in DP1 are shown in Table 2. High intake of vegetables, fruits, fish and small fish, natto (fermented soybeans), and deep fried tofu was associated with DP1.

**Table 2.** Factor loadings of food items in dietary pattern 1

Food items	Factor loadings^a^
Rice	0.041
Bread	0.008
Noodle	−0.014
Bread & margarine	−0.003
Bread & butter	0.073
Milk	0.337
Yogurt	0.337
Miso	0.326
Chilled tofu & toppings, boiled tofu	0.294
Natto (fermented soybeans), soya beans (other cooked beans)	0.455
Egg	0.223
Chicken	0.139
Beef, pork	0.216
Liver	0.045
Ham, sausage, salami, bacon	0.144
Fish (eg, sashimi, boiled fish, and grilled fish)	0.470
Small fish eaten with its bone	0.473
Canned tuna	0.146
Squid, shrimp, crab, octopus	0.173
Shellfish (eg, clam and oyster)	0.190
Cod roe, salmon roe	0.101
Fish paste cake, steamed seasoned fish paste	0.263
Deep-fried tofu with thinly sliced vegetables, deep-fried bean curd, deep-fried tofu	0.440
Potato, taro, sweet potato	0.599
Pumpkin	0.586
Carrot	0.650
Broccoli	0.491
Green leafy vegetables (eg, spinach, Japanese mustard spinach, and edible chrysanthemum)	0.702
Green & yellow vegetables (eg, green pepper and green beans)	0.602
Cabbage	0.531
Radish (boiled & grated radish)	0.558
Dried radish	0.330
Burdock, bamboo shoots	0.399
Other light-colored vegetables (eg, cucumber, onion, bean sprouts, Chinese cabbage, and lettuce)	0.625
Mushroom (eg, shiitake, enoki, and shimeji)	0.573
Seaweed (eg, hijiki and kelp)	0.476
Mayonnaise (include potato salad)	0.083
Deep fried food	0.098
Stir fry food (dish made with small amount of oil)	0.318
Mandarin orange, orange, grapefruit	0.495
Other fruits (eg, strawberry, kiwi, apple, watermelon)	0.564
Peanuts, almond	0.280
Western confectionary (eg, cake and creampuff)	0.170
Japanese confectionary (eg, steamed bun)	0.348
Green tea	0.283
Coffee	0.057

^a^Factor loadings represent the correlation of each food item with dietary pattern 1 score.

The prevalence rate of MetS was 18.1% in men and 12.8% in women, which was similar to that reported for another Japanese population by Arai et al^35^ (19% in men and 9% in women aged 40–64 years old). Baseline characteristics of the total study participants and subgroups according to the quartiles of DP1 score are shown in Table 3. Subjects with higher dietary pattern scores were more likely to be female, older, non-smokers, drank less alcohol, had higher energy intake, and participated more in leisure time physical activity. They also had lower BMI, waist circumference, and serum triglycerides, and higher serum HDL-cholesterol. The nutrients used as response variables (K, Ca, vitamin D, vitamin C, IDF, and carotene) were positively related to DP1 score.

**Table 3.** Baseline characteristics of the study participants based on the quartiles of the dietary pattern 1 score

	Total participants	Quartiles of dietary pattern score				*P*

		Q1	Q2	Q3	Q4	
Age, years^a^	53 (44, 61)	48 (41, 57)	51 (42, 59)	54 (45, 62)	59 (50, 63)	<0.0001
Sex^b^						
Male	537 (49.2)	175 (64.1)	150 (54.9)	126 (46.2)	86 (31.5)	<0.0001
Female	555 (50.8)	98 (35.9)	123 (45.1)	147 (53.8)	187 (68.5)	
BMI, kg/m^2 a^	22.8 (20.9, 25.1)	23.2 (21.5, 25.9)	22.9 (20.9, 25.3)	22.7 (20.7, 24.8)	22.1 (20.4, 24.1)	0.0002
Smoking^b^						
current	182 (16.7)	73 (26.7)	59 (21.6)	30 (11.0)	20 (7.3)	<0.0001
past	272 (24.9)	77 (28.2)	71 (26.0)	72 (26.4)	52 (19.0)	
never	638 (58.4)	123 (45.1)	143 (52.4)	171 (62.6)	201 (73.6)	
Drinking^c^						
current	598 (54.8)	177 (64.8)	149 (54.6)	143 (52.4)	129 (47.3)	0.0020
past	18 (1.7)	4 (1.5)	6 (2.2)	5 (1.8)	3 (1.1)	
never	476 (43.6)	92 (33.7)	118 (43.2)	125 (45.8)	141 (51.6)	
Total energy intake, kcal/day^a^	1657 (1472, 1884)	1606 (1387, 1851)	1664 (1479, 1886)	1645 (1485, 1892)	1696 (1531, 1885)	0.0021
Physical activity, MET-hours/week^a^	6.1 (1.2, 18.6)	3.0 (0.0, 8.9)	5.1 (1.2, 17.9)	7.9 (1.2, 21.3)	11.4 (3.0, 28.5)	<0.0001
Waist circumference, cm^a^	82.0 (75.0, 88.5)	83.0 (76.0, 90.0)	83.0 (76.0, 89.0)	82.0 (75.5, 88.0)	79.0 (74.0, 86.0)	0.0002
Systolic blood pressure, mm Hg^a^	122 (110, 134)	122 (110, 134)	120 (108, 134)	122 (112, 133)	124 (112, 136)	0.1799
Diastolic blood pressure, mm Hg^a^	74 (66, 82)	76 (67, 84)	73 (65, 81)	74 (66, 81)	74 (68, 84)	0.3162
Triglycerides, mg/dL^a^	87 (62, 126)	94 (66, 143)	92 (64, 134)	87 (62, 123)	77 (56, 113)	0.0002
HDL cholesterol, mg/dL^a^	59 (48, 69.5)	55 (46, 68)	57 (47, 66)	60 (49, 70)	63 (52, 74)	<0.0001
Fasting glucose, mg/dL^a^	92 (87, 99)	93 (88, 101)	92 (86, 98)	92 (87, 99)	91 (87, 97)	0.1939
Prevalence of metabolic syndrome^b^	168 (15)	55 (20)	37 (14)	39 (14)	37 (14)	0.093
**Nutrients intake**^a^						
K, mg/day	2042 (1771, 2342)	1636 (1492, 1814)	1931 (1758, 2092)	2121 (1994, 2293)	2580 (2346, 2807)	<0.0001
Ca, mg/day	476 (387, 587)	372 (329, 449)	441 (383, 519)	512 (416, 597)	610 (524, 708)	<0.0001
Vitamin D, µg/day	5.2 (4.5, 7.8)	4.6 (4.0, 4.9)	4.9 (4.5, 6.9)	7.0 (4.7, 8.0)	7.7 (5.8, 10.0)	<0.0001
Vitamin C, mg/day	86 (66, 111)	60 (52, 71)	77 (64, 90)	97 (80, 111)	124 (109, 148)	<0.0001
IDF, g/day	7.1 (6.0, 8.6)	5.6 (5.1, 6.1)	6.6 (6.0, 7.1)	7.6 (7.0, 8.4)	10.0 (8.9, 11.2)	<0.0001
Carotene, µg/day	2717 (2114, 3520)	1913 (1740, 2114)	2435 (2114, 2912)	2917 (2518, 3438)	4244 (3520, 5113)	<0.0001

BMI, body mass index; Ca, calcium; IDF, insoluble dietary fiber; K, potassium; MET, metabolic equivalent.

^a^Median (25%, 75%).

^b^Number (%).

Differences were analyzed using the Kruskal-Wallis test^a^, chi-square test^b^, or Fisher’s exact test^c^.

Logistic regression analysis showed that the DP1 score was negatively associated with the prevalence of MetS in model 1 adjusted for sex and age (OR 1.00, 0.59, 0.57, and 0.50 for quartiles 1–4, respectively; *P*_trend_ = 0.007) and model 2 adjusted for sex, age, energy intake, physical activity, smoking and drinking habit, and recruitment group (OR 1.00, 0.58, 0.60, and 0.52 for quartiles 1–4, respectively; *P*_trend_ = 0.02, Table 4). However, in the logistic regression analysis between DP1 and each component of MetS, significant inverse relationships could be found only between DP1 and blood pressure (OR 1.00, 0.64, 0.49, and 0.45 for quartiles 1–4, respectively; *P*_trend_ = 0.0002) and between DP1 and blood glucose level (OR 1.00, 0.66, 0.63, and 0.54 for quartiles 1–4, respectively; *P*_trend_ = 0.02), in model 2 (Table 4). The association between DP1 and abdominal obesity was of borderline significance (OR 1.00, 1.06, 0.93, and 0.70 for quartiles 1–4, respectively; *P*_trend_ = 0.07). There was no significant effect modification by sex on the dose-response relationship between DP1 score and MetS or its components.

**Table 4.** Associations of dietary pattern 1 score with MetS and its components

	Q1	Q2		Q3		Q4		*P*_trend_
			
	OR	OR	95% CI	OR	95% CI	OR	95% CI	
Metabolic syndrome								
Model 1^a^	1	0.59	0.37–0.94	0.57	0.36–0.92	0.50	0.30–0.82	0.007
Model 2^b^	1	0.58	0.36–0.94	0.60	0.37–0.97	0.52	0.30–0.89	0.021
Waist circumference								
Model 1^a^	1	1.02	0.71–1.45	0.86	0.60–1.24	0.63	0.43–0.93	0.013
Model 2^b^	1	1.06	0.73–1.52	0.93	0.64–1.36	0.70	0.46–1.04	0.066
Elevated triglycerides								
Model 1^a^	1	0.82	0.54–1.26	0.75	0.48–1.17	0.58	0.35–0.95	0.029
Model 2^b^	1	0.84	0.54–1.31	0.80	0.50–1.28	0.67	0.39–1.15	0.149
Blood pressure								
Model 1^a^	1	0.70	0.48–1.01	0.57	0.39–0.83	0.55	0.37–0.81	0.002
Model 2^b^	1	0.64	0.44–0.95	0.49	0.32–0.73	0.45	0.29–0.70	0.0002
Low HDL cholesterol								
Model 1^a^	1	1.36	0.83–2.25	1.14	0.68–1.91	0.74	0.42–1.32	0.250
Model 2^b^	1	1.27	0.76–2.14	1.17	0.68–2.03	0.72	0.38–1.36	0.311
Fasting glucose								
Model 1^a^	1	0.69	0.46–1.04	0.63	0.41–0.96	0.57	0.36–0.90	0.011
Model 2^b^	1	0.66	0.43–1.02	0.63	0.40–1.00	0.54	0.33–0.89	0.016

CI, confidence interval; HDL, high-density lipoprotein; OR, odds ratio.

Multivariate logistic regression analysis was performed to evaluate the associations of dietary pattern with MetS and its components.

Tests for trend across quartiles were performed by using ordinal categorical variables and likelihood ratio test.

^a^Adjusted for age and sex.

^b^Adjusted for age, sex, physical activity, energy intake, smoking habit, drinking habit, and recruitment group.

Since the proportions of men and women were different among the four groups classified according to the DP1 score, logistic regression analysis was performed using sex-specific cut-off points for DP1 score and covariates (eTable 1). The results did not differ greatly with the model 2 shown in Table 4.

## DISCUSSION

We identified the dietary pattern that was related to lower prevalence of MetS and inversely associated with separate components of MetS—blood pressure and blood glucose level—in a middle-aged Japanese population using the RRR method, independent of potential confounding variables. In the current study, we used K, Ca, vitamin D, vitamin C, IDF, and carotene as response variables. As a result, extracted dietary pattern was positively correlated with high intake of vegetables, fruits, fish and small fish, and soy products.

Many studies have examined the association between dietary patterns and MetS. Studies from Japan,^36^ the United States,^8^ Mexico,^37^ and Iran^38^ reported no association between healthy or prudent dietary pattern and MetS. In contrast, findings from the ATTICA Study,^39^ using principal component analysis, showed that consumption of cereals, vegetables, fish, legumes, and fruits were associated with reduced prevalence of MetS. The Bogalusa Heart Study^40^ showed similar results for the relation between a prudent dietary pattern and MetS; however, the association became insignificant after additionally adjusting for BMI. DP1 from our study shared similar components with the dietary patterns in prior studies, which included vegetables,^39^^–^^41^ fruits,^39^^,^^40^ and fish.^39^^,^^41^ The explained variance in food intake of DP1 in our study (14.3%) did not greatly differ from the explained variance of the ATTICA study,^39^ Korean Study,^41^ and Bogalusa Heart Study^40^ (19.7%, 5.7%, and 12%, respectively). However, in our study, the variance of nutrients explained by DP1 was quite high at 60.1%. This was because dietary patterns derived from RRR are driven by particular response variables instead of food intake.

The significant association between DP1 and blood pressure may be attributed to K, IDF, and Ca. A previous meta-analysis of intervention studies reported that high intake of dietary K was inversely related with blood pressure.^42^ Several mechanisms have been proposed regarding the effects of K on blood pressure—increased sodium excretion in urine,^43^ reduced activity of sympathetic nerves,^44^ and lower pressor response to norepinephrine and angiotensin II.^45^ Dietary intake of K comes from tofu, natto, fruits, and vegetables.^46^^,^^47^ Earlier prospective cohort studies suggested the beneficial effects of dietary fiber on blood pressure.^48^^–^^50^ Although the relationship between insoluble dietary fiber and blood pressure has been less explored in-depth, Lairon et al^51^ reported that consumption of foods high in total dietary fiber and IDF can help to reduce the prevalence of hypertension, while there was no association found for soluble dietary fiber. Aljuraiban et al^52^ also stated that higher consumption of fiber, particularly IDF (not soluble dietary fiber), may lower blood pressure. Furthermore, it has been suggested that the beneficial effect of dietary fiber on blood pressure is due to the enhancement of insulin sensitivity, which possibly improves endothelial function.^53^ Prior investigations showed dietary intake of Ca, but not Ca supplements, was inversely related with risk of hypertension.^54^^,^^55^ On the other hand, a meta-analysis of randomized controlled trials reported that Ca supplementation could reduce systolic and diastolic blood pressure. The decrease in blood pressure was more pronounced when the initial Ca intake was equal to or lower than 800 mg/day.^56^ Ca may work together with K, sodium, and magnesium to decrease blood pressure by providing ionic balance in the vascular membrane, as well as vasodilatation.^57^

The inverse relation between DP1 and fasting glucose was presumably because of IDF, Ca, carotene, and vitamin C. The effect of IDF on the concentration of glucose in the blood is possibly related to insulin sensitivity enhancement.^58^ Although we did not include soluble dietary fiber in the response variables, it is necessary to mention the importance of soluble dietary fiber in glucose metabolism, since natural high-fiber foods contain both soluble and insoluble dietary fibers in various amounts. Soluble dietary fiber may lower the blood glucose level by forming a gel in the stomach that delays gastric emptying. Thus, glucose absorption and postprandial blood insulin levels decrease.^59^ Drouillet et al^60^ found no association between Ca and fasting glucose. In contrast, greater intake of Ca was found to be inversely related with fasting blood glucose in Dutch^61^ and Korean studies.^62^ In our study, abundant sources of Ca could be found in small fish eaten with the bones, natto, and tofu. According to Ylönen et al,^63^ intakes of dietary α-carotene, β-carotene, and lycopene can have beneficial effects on glucose metabolism in subjects at high risk for type 2 diabetes mellitus. However, the mechanism of this advantageous association is largely unknown. In addition to IDF, Ca, and carotene, vitamin C supplementation was reported to significantly reduce the fasting blood sugar level.^64^ In diabetic patients given vitamin C and metformin orally, glycemic control was improved presumably because of the antioxidant effects on β cells.^65^

The inverse association between DP1 and abdominal obesity was of borderline significance (*P*_trend_ = 0.07). A prior study showed that increased consumption of fruit and vegetables was negatively associated with central obesity.^66^ A possible explanation of this advantageous effect is the fiber content in fruit and vegetables, which may result in reduced energy intake by lowering food energy density and maintaining satiation.^67^

The significant association between DP1 and MetS may be also caused by other nutrients that are present in foods with high correlations with DP1, but not used as response variables in our analysis. For example, vegetables and fruits may also contain magnesium,^11^^,^^68^ which can lower the blood pressure^69^ and fasting blood glucose^70^; vitamin E,^71^ which can have a favorable effect on blood pressure; and vitamin K,^72^ which is associated with reduced waist circumference.^73^ Soy products are also sources of soy protein^74^ and isoflavones,^75^ which may have a favorable effect on blood glucose, body weight, and fasting insulin.^76^^,^^77^

We found no significant associations between DP1 and two components of MetS—triglycerides and HDL cholesterol (Table 4). One of the nutrients reported to be associated with triglycerides and HDL cholesterol is n-3 polyunsaturated fatty acids (n-3 PUFA).^78^ However, the correlation between n-3 PUFA and DP1 was weaker (0.46) in our study compared with correlations between DP1 and other nutrients chosen as response variables. This may explain the reason why no significant association was seen between these two MetS components and DP1.

The strengths of the present study are the usage of a validated FFQ and adjustment for potential confounding variables in the analyses. To the best of our knowledge, this is the first study to examine the relationship between dietary pattern extracted using the RRR method, based on selected nutrients, and MetS in Japan. An advantage of using the RRR method was to overcome the high correlations among nutrients. In fact, there were significant inverse associations of K, Ca, vitamin C, IDF, and carotene with the prevalence of MetS. However, there were strong positive correlations, especially between IDF and carotene (Spearman *r* = 0.78) and vitamin C and IDF (*r* = 0.72), and it was difficult to adjust for the mutual effects in logistic regression analysis. A number of limitations in this study are worth mentioning. First, rate of participation was low, especially in the second group of persons who responded to our leaflets. So, it is not known whether the study subjects were representative of the general population, and whether the results are applicable to the general population of Tokushima Prefecture or all over Japan. Second, since the RRR analysis depends on the data at hand, the results from this analysis are not entirely reproducible by other studies. Third, the RRR dietary pattern is not based on dietary behavior, so the application value of this pattern in reality is unclear.^79^^,^^80^ Nevertheless, when we compared the dietary pattern extracted by RRR and PCA, factor loadings of DP1 were not greatly different from the first component of PCA, probably because of the selection of response variables (data not shown). Thus, we assume that the RRR derived dietary pattern used in this study may explain the actual dietary pattern of this population. Fourth, we used a cross-sectional study design, so the temporal relationship between DP1 score and MetS is unclear. Finally, dietary intake and lifestyle factors data were gathered using a self-administered questionnaire. As a result, random measurement errors may be inevitable.

In conclusion, a dietary pattern characterized by high intake of vegetables, fruits, fish and small fish, natto, and deep fried tofu was associated with reduced prevalence of MetS and separate components of MetS—blood pressure and blood glucose level—in a middle-aged Japanese population. This result can be used for formulating dietary guidelines to promote healthy dietary intake in the Japanese population. In the future, prospective studies are needed to clarify the causal relationship of this result.

## APPENDIX A. SUPPLEMENTARY DATA

eTable 1. Associations of dietary pattern 1 with metabolic syndrome and each component
