# Supplementary material for: Nutrient-Derived Dietary Patterns and Their Association With Metabolic Syndrome in a Japanese Population
Source: J Epidemiol. 2018 Apr 5;28(4):194–201. doi: 10.2188/jea.JE20170010 (PMC5865010; doi:10.2188/jea.JE20170010)
Supplement: Supplementary file 1 [file je-28-194-s001.pdf]

eTable 1. Associations of dietary pattern 1 with metabolic syndrome and each component

	Q1	Q2		Q3		Q4		P_{trend}
	OR	OR	95% CI	OR	95% CI	OR	95% CI	
Metabolic syndrome								
Model 1[a]	1	0.68	0.43-1.08	0.59	0.37-0.94	0.55	0.34-0.88	0.010
Model 2[b]	1	0.66	0.41-1.07	0.58	0.35-0.95	0.52	0.31-0.88	0.013
Waist circumference								
Model 1[a]	1	0.87	0.62-1.24	0.81	0.57-1.16	0.60	0.42-0.88	0.008
Model 2[b]	1	0.87	0.61-1.25	0.83	0.58-1.21	0.59	0.39-0.89	0.015
Elevated triglycerides								
Model 1[a]	1	0.60	0.38-0.94	0.83	0.54-1.27	0.51	0.32-0.82	0.026
Model 2[b]	1	0.63	0.39-0.99	0.86	0.55-1.35	0.54	0.32-0.90	0.067
Blood pressure								
Model 1[a]	1	0.75	0.52-1.08	0.64	0.44-0.93	0.58	0.39-0.84	0.003
Model 2[b]	1	0.68	0.46-1.00	0.55	0.37-0.82	0.48	0.31-0.74	0.0004
Low HDL cholesterol								
Model 1[a]	1	1.29	0.78-2.14	1.31	0.79-2.17	0.75	0.43-1.33	0.389
Model 2[b]	1	1.24	0.73-2.09	1.28	0.75-2.18	0.67	0.36-1.24	0.270
Fasting glucose								
Model 1[a]	1	0.98	0.65-1.48	0.65	0.42-1.00	0.69	0.45-1.06	0.029
Model 2[b]	1	1.02	0.65-1.58	0.67	0.42-1.07	0.73	0.45-1.20	0.087

CI, confidence interval; HDL, high-density lipoprotein; OR, odds ratio.

Multivariate logistic regression analysis was performed to evaluate the associations of dietary pattern with MetS and its components.

Sex-specific cut-off points for the dietary pattern 1 score, total energy intake, and physical activity were used.

Tests for trend across quartiles were performed by using ordinal categorical variables and likelihood ratio test.

[a]Adjusted for age and sex.

[b]Adjusted for age, sex, physical activity, energy intake, smoking habit, drinking habit, and recruitment group.
